# Validation of the EORTC QLQ-C30 and QLQ-BN20, including WHO performance status interrater reliability, for evaluation of patients with intracranial meningiomas

**DOI:** 10.1093/nop/npae125

**Published:** 2024-12-24

**Authors:** Robert F Nilsson, Erik Ström, A Tommy Bergenheim, Rickard L Sjöberg, Peter Lindvall, Klas Holmgren

**Affiliations:** Department of Clinical Science—Neurosciences, Umeå University, Umeå, Sweden; Department of Clinical Science—Neurosciences, Umeå University, Umeå, Sweden; Department of Clinical Science—Neurosciences, Umeå University, Umeå, Sweden; Department of Clinical Science—Neurosciences, Umeå University, Umeå, Sweden; Department of Clinical Science—Neurosciences, Umeå University, Umeå, Sweden; Department of Clinical Science—Neurosciences, Umeå University, Umeå, Sweden

**Keywords:** meningioma, quality of life, validation

## Abstract

**Background:**

The EORTC questionnaires QLQ-C30 and QLQ-BN20 are commonly used to evaluate health-related quality of life in patients with meningiomas but have not undergone a disease-specific validation. The study aimed to address this issue and to determine the interrater reliability of WHO performance status (PS) assessments in these patients.

**Methods:**

This population-based study included prospectively enrolled intracranial meningiomas treated at Umeå University Hospital between October 14, 2010, and December 31, 2021, followed up until March 30, 2023. Patients were assessed by the EORTC questionnaires before and at 3 months after surgery. WHO PS categorized as high (0–1) or low (2–5) were evaluated for interrater reliability and used together with sick-leave status to determine the questionnaires’ clinical validity. Remaining psychometric properties of the questionnaires were analyzed by conventional methods.

**Results:**

Of 513 eligible surgeries, 454 (88.5%) had responded to at least 1 questionnaire. WHO PS interrater agreement was 94.4%. The EORTC questionnaires’ ability to distinguish between clinically distinct groups was high. Items correlated better with their own scale than others (most *r* > 0.70). Items measuring various aspects of the same construct showed good internal consistency (nearly all α > 0.70). Questionnaire responsiveness to symptom changes over time was acceptable. Several scales displayed floor and ceiling effects.

**Conclusions:**

The EORTC QLQ-C30 and QLQ-BN20 are overall valid instruments to evaluate patients with intracranial meningiomas but require awareness of certain limitations when specific functions and symptoms are addressed. WHO PS assessments can be applied to meningioma patients with high reproducibility between observers.

**Key Points**

• Population-based validation of the EORTC QLQ-C30 and BN20 for intracranial meningiomas.
• Limitations and validity in evaluation of meningiomas with the EORTC QLQ-C30 and BN20.

**Importance of the Study**

The EORTC questionnaires QLQ-C30 and brain cancer module QLQ-BN20 are among the most widespread and advocated instruments to assess health-related quality of life in patients with meningiomas but they have not been fully validated. This large and disease-specific population-based study addressed this issue and demonstrates that the questionnaires are generally valid for evaluation of patients with intracranial meningiomas but also that researchers need to recognize a few limitations when certain outcomes are assessed. Specifically, some items and scales may be insufficient in capturing the full range of severity in meningioma patients and suggest additional analyses or complementary instruments are likely to be required when these symptoms and functions are of particular interest. Moreover, WHO performance status judged by 2 independent observers demonstrated a substantial level of agreement when categorized as either high or low and could be a reproducible measure for assessment of patients with intracranial meningiomas.

Meningiomas are the most prevalent of intracranial tumours^1^ and may cause a wide spectrum of symptoms, ranging from neurological disabilities and seizures to psychiatric disorders and cognitive deficits. Patients also frequently experience nonspecific symptoms such as fatigue, sleep disturbance, and headache. While neurosurgical resection, in addition to tumor removal, aims to alleviate symptoms and increase well-being, many patients nonetheless sustain disabilities several years after treatment.^2,3^ In addition, recent studies have demonstrated that patients treated for meningiomas report considerable limitations in long-term health-related quality of life (HRQoL).^3,4^ However, large population-based reports on long-term HRQoL for patients with meningiomas remain scarce, in particular, studies using validated instruments.^2^

The European Organization for the Research and Treatment of Cancer Core Quality of Life Questionnaire (EORTC QLQ-C30) is a generally well-established tool for assessing HRQoL and symptoms among cancer patients.^5^ Combined with the brain cancer module QLQ-BN20, the instruments capture a wide range of functional deficits and symptoms specific for brain cancer patients.^6^ While specific tools to assess HRQoL in patients with meningiomas have been suggested,^7^ the EORTC forms remain among the most widely applied in meningioma research and were considered most suitable in a review of questionnaires to evaluate HRQoL in patients with meningiomas specifically.^2^ Although the EORTC instruments have been validated in patients with brain tumors of different histological subtypes,^8–13^ most have focused exclusively on malignant tumors such as gliomas or metastases, for which management, treatment, and clinical course differ substantially. An adequately powered and disease-specific validation of the EORTC questionnaires to evaluate HRQoL in patients with meningiomas has thus become increasingly warranted.^2,3,14^

The present study aims to validate the EORTC QLQ-C30 and BN20 questionnaires for assessment of HRQoL and symptoms in patients specifically with intracranial meningiomas. To anchor the questionnaires to a preexisting measure of global functional status, the WHO performance status (PS) grading system, as well as working capacity estimated via sick-leave statistics, were used. Moreover, as the interrater agreement of PS ascertainment between healthcare professionals may in some settings be subject to variability,^15,16^ and its applicability to meningiomas patients unestablished, a corresponding supplemental analysis was also included.

## Methods

### Study Design

This is a population-based validation study approved by the Regional Ethics Committee. The study cohort was composed of prospectively enrolled patients with intracranial meningiomas eligible for neurosurgical treatment at Umeå University Hospital between October 14, 2010, and December 31, 2021, with follow-up until March 30, 2023. Written informed consent was obtained from all participants before study inclusion. HRQoL was assessed by means of the EORTC core questionnaire (QLQ-C30) and brain-specific module (QLQ-BN20) prior to surgery, and at 3 months after treatment in connection to a routine outpatient follow-up visit. All patients who had undergone craniotomy for a histopathologically confirmed intracranial meningioma and completed at least 1 of the 2 questionnaires were included. Baseline characteristics were collected by scrutiny of medical records. All patients’ functional status were estimated according to the WHO PS grading system by retrospective chart review. The WHO PS estimations were matched to the time point of each HRQoL assessment and ascertained by 2 separate investigators (R.N. and K.H.) for all patients treated prior to December 31, 2020, and categorized as having either a high (PS 0–1) or a low (PS 2–5) PS. As patients treated during the year 2021 were included for study at a later time point, and the number of observations to estimate interrater reliability was already considered sufficient, WHO PS for these patients was determined by one of the investigators exclusively (K.H.). Data on sick leave was collected from the Swedish Social Insurance Agency for patients aged 18–60 years, and patients were considered being on sick leave if receiving any of the following financial aids: sickness benefit, preventive sickness benefit, rehabilitation benefit, occupational injury sickness benefit, early retirement benefit, sickness allowance, sickness compensation, or activity compensation. Patients’ sick-leave status was dichotomized as being on sick leave (fully or partially), or not, and was determined matched to the date on which the questionnaire had been completed for each patient individually.

### Instrument

The EORTC QLQ-C30 questionnaire consists of 30 items organized into 5 functional scales (physical, role, emotional, cognitive, and social functioning), 3 multi-item symptom scales (fatigue, nausea and vomiting, and pain), 6 single-item symptom scales (dyspnea, insomnia, appetite loss, constipation, diarrhea, financial difficulties) as well as a global health status/QoL scale. The items are scored on a 4-point Likert scale ranging from “not at all” (1) to “very much” (4), except for 2 items assessing global health status/QoL scored on a 7-point linear analog scale.

The EORTC QLQ-BN20 is a supplemental questionnaire to be used in addition to the QLQ-C30 in patients with brain tumors and comprises 20 complementary items, each scored on the same 4-point scale used in the QLQ-C30. The QLQ-BN20 is organized into 4 multi-item symptom scales (future uncertainty, visual disorder, motor dysfunction, and communication deficit) and 7 single-item symptom scales (headaches, seizures, drowsiness, hair loss, itchy skin, weakness of legs, and bladder control).

Raw scores were calculated and linearly transformed into scale scores ranging from 0 to 100 for all scales in the questionnaires according to the EORTC QLQ-C30 and QLQ-BN20 respective scoring manuals.^17^ Higher scores on a functional scale and the global health status/QoL scale indicate higher levels of functioning and health status/QoL, respectively, whereas higher scores on a symptom scale represent more pronounced symptoms.

### Validity

Psychometric properties of the questionnaires were evaluated by assessment of floor and ceiling effects, convergent and discriminant validity, internal consistency, clinical validity, responsiveness, and construct validity. To clarify, floor and ceiling effects are present when a substantial number of respondents report the lowest or highest scores possible on a specific item or scale, which may indicate that the symptom or experience measured could be of limited relevance to the patients or lack sensitivity to different levels of severity. Convergent validity determines the relationship between a scale and its associated items, whereas discriminant validity addresses the relationship between a scale and other questionnaire items that are considered conceptually unrelated. Low convergent validity would, for example, indicate that items do not correlate with their associated scale, and that the scope of the items is likely too wide or unspecific, while low discriminant validity would denote too much association between items and scales that are conceptually unrelated. Internal consistency measures the correlation between items within the same multi-item scale (ie, that items supposed to be associated display converging scores). Clinical validity and responsiveness evaluate the questionnaires’ ability to distinguish between well-defined groups of patients based on their clinical condition. Shortcomings in clinical validity would indicate that the instruments cannot differentiate between patients whose clinical status differs significantly, while low responsiveness would suggest a lack of ability to capture changes over time when the same construct is measured at different time points. Construct validity refers to how well an item or scale measures the specific concept it is intended to evaluate (eg, if the Physical function scale actually reflects the respondent’s physical function) and, in addition to convergent and discriminant validity, was evaluated by measuring the level of association between different scales of the QLQ-C30 and QLQ-BN20. Strong associations between conceptually different scales could for instance indicate that they reflect constructs that they are not intended to.^18,19^

### Statistical Analyses

The interrater reliability of WHO PS assessments was analyzed and presented with level of agreement as well as Cohen’s kappa coefficient (κ).^20^ While the latter to some extent constitutes an arbitrary measure, interpretation adhered to generally established guidelines, in which a value of 0–0.6 would denote poor to moderate agreement, 0.6–0.8 would suggest substantial conformity, whereas a value greater than or equal to 0.8 would indicate near-perfect agreement.^21^

To ensure that the study was sufficiently powered to validate the EORTC questionnaires, a sample size calculation was performed prior to study start. In line with previous validation reports, a minimum of 10 observations per item was deemed sufficient,^8,22^ and a sample size of at least 500 completed forms was thus considered necessary to evaluate the total of 50 items covered by the 2 EORTC questionnaires. It was subsequently stipulated that at least 50 patients with intracranial meningiomas had undergone surgery at the study hospital per year, hence corresponding to 100 questionnaires if all eligible patients had chosen to participate and completed both the preoperative and the postoperative forms. As the study period spanned over more than 11 years, at least 550 patients were assumed to have had been operated on during that time, resulting in a potential 1100 questionnaires having been distributed. A response rate as low as 50% would thus amount to 550 questionnaires completed and still be able to account for substantial missing and unforeseen dropouts. As the response rate was expected to be significantly higher than 50%, the number of completed questionnaires was thus expected to meet and likely exceed the required sample size of 500.

Mean value and standard deviation (SD) were calculated for all EORTC questionnaire scale scores. Item floor and ceiling effects were considered present if >15% of respondents had reported scores on the lower or upper limit.^23^ Convergent validity was analyzed by means of Spearman correlation between each item and its own scale, for which a correlation coefficient (*r*) > 0.40 was considered significant. Discriminant validity was assessed by comparing the interconnectedness between items and scales, wherein an item was expected to correlate better (>2 SE) with its own scale, as when compared to the other scales. Internal consistency among items in the same multi-item scale was calculated with Cronbach’s alpha (α), where α > 0.70 was deemed acceptable.^24^ Clinical validity was determined based on the questionnaires’ ability to distinguish between known groups of patients, that is, groups of patients categorized as high or low WHO PS, and between those with working capacity versus those on sick leave, respectively. Differences between groups were analyzed with the Mann–Whitney *U* test. Low WHO PS (PS 2–5) was hypothesized to be associated with reduced physical functioning, fatigue, insomnia, visual disorder, motor dysfunction, drowsiness, weakness of legs, and reduced global health status/QoL. Sick leave was hypothesized to be associated with reduced physical, emotional, and cognitive functioning, as well as pain, insomnia, financial difficulties, future uncertainty, visual disorder, motor dysfunction, headache, seizures, drowsiness, weakness of legs, and reduced global health status/QoL. Furthermore, to assess responsiveness of the questionnaires to changes over time, patients were classified as having worsened (indicated by a shift in WHO PS from high [0–1] to low [2–5]), or having remained stable or improved (denoted by an unchanged or any shift to a higher WHO PS). Shifts in PS were then compared with changes according to the questionnaires (indicated by a worsened, stable, or improved scale score) during the same time period using Fisher’s exact test. Finally, evaluation of construct validity was complemented by analyzing Spearman correlation between the scales of QLQ-C30 with those of QLQ-BN20, for which *r *> 0.40 was considered a meaningful correlation. While some specific constructs are included in both questionnaires, a few correlations were expected, whereas the overall aim of the 2 questionnaires is to supplement each other, and should thus result in mostly low to moderate correlations between scales. In line with previous validation studies, only complete case analyses were included.^8,10^ All statistical analyses were conducted using the computer software Stata/BE version 17 (StataCorp LLC). Significance level was set at *P* < .05.

## Results

### Baseline Characteristics

**Figure 1.**
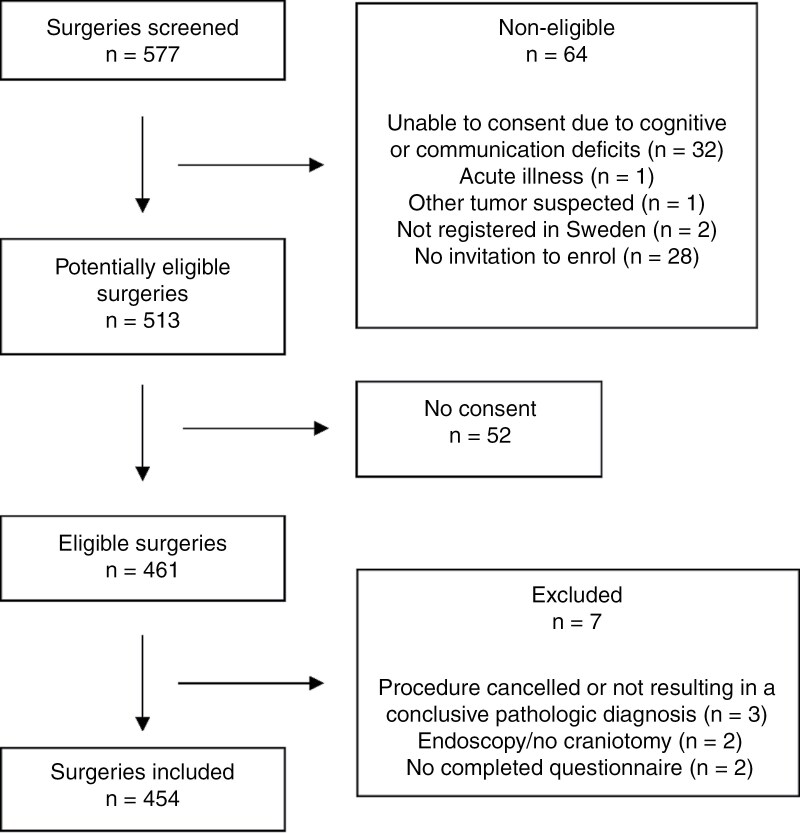
Flow chart depicting selection of surgeries and exclusion criteria.

Of a total of 577 surgeries screened, 513 were deemed eligible and had been queried for study inclusion, out of which 52 had declined study participation, with a total of 454 remaining and included in the study cohort. Patient selection and exclusion criteria are shown in Figure 1. Baseline characteristics including age, sex, tumor location, WHO grade, number of tumors resected during each surgery, and numerical order of the operation are provided in Table 1.

**Table 1.** Patient Demographics and Baseline Characteristics

Age	*n* (%)
Median (IQR)	63 (52–71)
Sex	
Female	322 (70.9)
Male	132 (29.1)
Tumor location^a^	
Convexity	226 (48.2)
Anterior skull-base	70 (14.9)
Middle skull-base	92 (19.6)
Posterior skull-base	24 (5.1)
Tentorium	18 (3.8)
Falx	33 (7.1)
Pineal gland	1 (0.2)
Optic nerve	1 (0.2)
Intraventricular	4 (0.9)
WHO grade	
1	417 (91.9)
2	36 (7.9)
3	1 (0.2)
Number of tumors resected	
1	441 (97.1)
2	11 (2.4)
3	2 (0.4)
Numerical order^a^	
First	415 (88.5)
Reoperation	40 (8.5)
Second reoperation	12 (2.6)
Third reoperation	2 (0.4)

Abbreviations: WHO, World Health Organization.

^a^Total number = total number of tumors resected (some patients [*n* = 13] underwent resection of more than 1 tumor during the same operation).

### WHO PS Interrater Reliability

The interrater reliability analysis of WHO PS ascertainment indicated a substantial level of agreement (94.4%), κ = 0.76 (95% CI 0.69–0.83) (Table 2).

**Table 2.** Interrater Reliability of WHO PS Assessment

	K.H.		
R.N.	High PS	Low PS	Total
High PS	631	41	672
Low PS	1	79	80
Total	632	120	752
Agreement	94.4%		
Kappa (κ)	0.76 (95% CI 0.69–0.83)		
*P*	<.0001		

Abbreviations: WHO, World Health Organization; PS, performance status; R.N., first investigator; K.H., second investigator.

### EORTC Questionnaires’ Descriptive Statistics

A total of 441 (97%) preoperative and 326 (72%) postoperative EORTC questionnaires were completed. Mean values and SD for the QLQ-C30 and QLQ-BN20 scales, including floor and ceiling effects, as well as WHO PS and sick-leave status are shown in Table 3. Due to administrative failure, 60 patients (13.6%) had completed the intended preoperative questionnaire first after surgery and were therefore excluded from the analysis concerning preoperative scale scores. In addition, as scale scores could not be calculated in patients with single items missing, the total number of observations varied slightly between different scales. Several items and scales demonstrated floor and ceiling effects, reflecting that many patients experienced pronounced impairments or did not experience certain symptoms or loss of specific functions at all.

**Table 3.** Descriptive Statistics of EORTC Questionnaire Results, WHO Performance Status, and Sick-Leave Status

	Preoperatively				Postoperatively			
	Median (IQR)				Median (IQR)			
Number of days prior to/after surgery	8 (2–15)				96 (80–122)			

EORTC QLQ-C30	Obs	Mean (SD)	*N* (%) Floor	*N* (%) Ceiling	Obs	Mean (SD)	*N* (%) Floor	*N* (%) Ceiling
Global health status/QoL	377	56.6 (26.1)	16 (4.2)	24 (6.4)	322	65.1 (22.5)	3 (0.9)	34 (10.6)
Functional scales								
Physical functioning	375	80.1 (22.2)	1 (0.3)	107 (28.5)	317	78.7 (21.6)	3 (1.0)	66 (20.8)
Role functioning	378	64.2 (35.1)	41 (10.9)	134 (35.5)	322	66.3 (32.2)	25 (7.8)	111 (34.5)
Emotional functioning	378	66.8 (25.8)	8 (2.1)	52 (13.8)	319	76.3 (23.5)	5 (1.6)	91 (28.5)
Cognitive functioning	381	73.2 (27.6)	16 (4.2)	127 (33.3)	321	77.4 (24.4)	5 (1.6)	123 (38.3)
Social functioning	376	73.0 (29.4)	22 (5.9)	146 (38.8)	318	76.0 (27.6)	12 (3.8)	136 (42.8)
Symptom scales								
Fatigue	379	37.2 (27.6)	49 (12.9)	18 (4.8)	323	35.4 (25.6)	45 (13.9)	9 (2.8)
Nausea and vomiting	380	6.1 (14.1)	295 (77.6)	1 (0.3)	325	4.6 (11.5)	265 (81.5)	0 (0.0)
Pain	381	28.5 (30.7)	148 (38.9)	23 (6.0)	321	23.7 (27.6)	135 (42.1)	9 (2.8)
Dyspnea	379	18.3 (25.5)	223 (58.8)	12 (3.2)	324	20.8 (24.5)	166 (51.2)	5 (1.5)
Insomnia	380	35.3 (33.5)	139 (36.6)	42 (11.1)	326	28.8 (29.8)	136 (41.7)	5 (1.5)
Appetite loss	381	9.6 (21.4)	305 (80.1)	5 (1.3)	326	10.7 (21.7)	249 (76.4)	5 (1.5)
Constipation	380	11.1 (22.5)	289 (76.1)	9 (2.4)	326	9.5 (19.6)	252 (77.3)	4 (1.2)
Diarrhea	379	6.6 (17.7)	323 (85.2)	4 (1.1)	324	7.0 (16.1)	266 (82.1)	1 (0.3)
Financial difficulties	378	14.3 (27.6)	282 (74.6)	17 (4.5)	322	15.0 (26.4)	226 (70.2)	12 (3.7)
EORTC QLQ-BN20								
Multi-item scales								
Future uncertainty	370	30.4 (23.4)	46 (12.4)	7 (1.9)	318	20.4 (20.4)	84 (26.4)	0 (0.0)
Visual disorder	376	15.7 (22.3)	191 (50.8)	5 (1.3)	321	13.1 (20.0)	181 (56.4)	2 (0.6)
Motor dysfunction	372	18.1 (24.3)	161 (43.3)	7 (1.9)	321	13.2 (20.4)	174 (54.2)	4 (1.3)
Communication deficit	376	13.2 (19.9)	197 (52.4)	4 (1.1)	322	11.1 (16.7)	176 (54.7)	1 (0.3)
Single-item scales								
Headaches	378	29.5 (32.4)	168 (44.4)	35 (9.3)	324	22.7 (27.9)	169 (52.1)	12 (3.7)
Seizures	375	5.2 (16.5)	334 (89.1)	3 (0.8)	323	2.0 (10.5)	309 (95.7)	2 (0.6)
Drowsiness	378	36.2 (30.9)	109 (28.8)	38 (10.1)	324	32.5 (28.5)	101 (31.2)	21 (6.5)
Hair loss	377	6.2 (19.2)	333 (88.3)	8 (2.1)	322	9.7 (23.0)	260 (80.8)	11 (3.4)
Itchy skin	376	8.8 (20.0)	303 (80.6)	5 (1.3)	323	10.9 (20.6)	238 (73.7)	5 (1.6)
Weakness of legs	373	14.3 (26.1)	265 (71.1)	16 (4.3)	322	12.3 (22.9)	234 (72.7)	7 (2.2)
Bladder control	378	15.4 (27.8)	264 (69.8)	23 (6.1)	324	11.1 (22.3)	247 (76.2)	5 (1.5)

WHO performance status		*N* (%)				*N* (%)		
High (PS 0–1)		316 (71.7)				253 (77.6)		
0		214 (48.5)				153 (47.0)		
1		102 (23.1)				100 (30.7)		
Low (PS 2–5)		125 (28.3)				73 (22.4)		
2		109 (24.7)				63 (19.3)		
3		16 (3.6)				10 (3.1)		
4		0 (0.0)				0 (0.0)		
5		0 (0.0)				0 (0.0)		
N/A (no pre- or postop form, respectively)		13				128		
Sick-leave status								
Fully or partially on sick leave		124 (36.5)				91 (39.2)		
Not on sick leave		216 (63.5)				141 (60.8)		

Abbreviations: EORTC, European Organization for the Research and Treatment of Cancer; QLQ-C30, Core Quality of Life Questionnaire; QLQ-BN20, Brain cancer-specific Quality of Life Questionnaire; QoL, quality of life; WHO, World Health Organization; PS, performance status; IQR, interquartile range; SD, standard deviation; Obs, observations; N/A, not applicable.

### Validity

The convergent validity of the questionnaires met the preset criteria |*r*| > 0.40 for item-own scale correlation except for item 5 (“Do you need help with eating, dressing, washing yourself, or using the toilet?”) and the Physical functioning scale (|*r*|* *= 0.35).

All items correlated better with their own scale compared to the other scales. Correlation coefficients were >2 SE higher for all item-own scale correlations compared to item-other scale correlations, and thus fulfilled the predetermined criterion for discriminant validity. Moreover, the internal consistency of the multi-item scales met the preset threshold (α* *> 0.70) for all scales except for Nausea and vomiting (α* *= 0.56) (Table 4).

**Table 4.** Convergent Validity, Discriminant Validity, and Internal Consistency

		Item-Own Scale Correlation		Item-Other Scale Mean Correlation		Internal Consistency
EORTC QLQ-C30	No. of Items in Scale	Observations	Spearman’s Rho	Spearman’s Rho	SE	Cronbach’s Alpha
Global health status/QoL	2	759	0.96 to 0.97	0.43 to 0.43		0.92
Functional scales						
Physical functioning	5	751	−0.89 to −0.35	0.18 to 0.37	0.01 to 0.03	0.84
Role functioning	2	760	−0.96 to −0.95	0.40 to 0.41	0.03 to 0.03	0.90
Emotional functioning	4	757	−0.87 to −0.75	0.31 to 0.35	0.02 to 0.03	0.88
Cognitive functioning	2	761	−0.88 to −0.86	0.35 to 0.39	0.02 to 0.03	0.73
Social functioning	2	753	−0.95 to −0.89	0.38 to 0.40	0.03 to 0.03	0.86
Symptom scales						
Fatigue	3	762	0.85 to 0.89	0.41 to 0.43	0.03 to 0.03	0.86
Nausea and vomiting	2	765	0.49 to 0.98	0.13 to 0.27	0.01 to 0.01	0.56
Pain	2	762	0.91 to 0.95	0.36 to 0.37	0.02 to 0.03	0.87
Dyspnea	1	762	1	0.3	0.02	
Insomnia	1	766	1	0.3	0.02	
Appetite loss	1	767	1	0.28	0.02	
Constipation	1	766	1	0.2	0.01	
Diarrhea	1	763	1	0.18	0.01	
Financial difficulties	1	760	1	0.28	0.02	
EORTC QLQ-BN20						
Multi-item scales						
Future uncertainty	4	746	0.54 to 0.87	0.18 to 0.35	0.02 to 0.03	0.79
Visual disorder	3	756	0.56 to 0.89	0.19 to 0.32	0.01 to 0.02	0.79
Motor dysfunction	3	752	0.68 to 0.87	0.24 to 0.36	0.02 to 0.03	0.80
Communication deficit	3	757	0.60 to 0.91	0.22 to 0.30	0.01 to 0.02	0.82
Single-item scales						
Headaches	1	761	1	0.28	0.02	
Seizures	1	757	1	0.15	0.01	
Drowsiness	1	761	1	0.38	0.03	
Hair loss	1	758	1	0.16	0.01	
Itchy skin	1	758	1	0.16	0.01	
Weakness of legs	1	754	1	0.31	0.02	
Bladder control	1	761	1	0.22	0.01	

Abbreviations: EORTC, European Organization for the Research and Treatment of Cancer; QLQ-C30, Core Quality of Life Questionnaire; QLQ-BN20, brain cancer-specific Quality of Life Questionnaire; QoL, quality of life; Obs, observations; SE, standard error.

All scale scores were significantly different between patients with high and low PS. Patients with high PS reported higher scores for the Global health status/QoL scale and Functional scales corresponding to higher health status/QoL and level of functioning, respectively, and lower scores for the symptom scales corresponding to a lower level of symptomatology. All results were thus in line with the clinical hypotheses stipulated a priori. For sick-leave status, scale scores differed significantly between those with working capacity and those on sick leave for all scales except Constipation, Diarrhea, Seizures, Hair loss, and Itchy skin. In similarity with patients with high PS, patients who were not on sick leave reported higher scores for the Global health status/QoL scale and Functional scales, and lower scores for the symptom scales. The results were thus all in agreement with the stipulated hypotheses except for the Seizures scale (Table 5).

**Table 5.** Clinical Validity and Responsiveness

EORTC QLQ-C30	WHO PS				Sick Leave				Worsened WHO PS		Stable or Improved WHO PS		
	Obs	High	Low	*P*	Obs	Yes	No	*P*	Obs	Mean (SD)	Obs	Mean (SD)	*P*
Global health status/QoL	653	67.3 (21.4)	38.9 (21.8)	<.01	285	51.1 (32.0)	72.8 (21.0)	<.01	22	−14.0 (29.7)	238	9.6 (23.7)	<.01
Functional scales													
Physical functioning	647	86.0 (15.7)	61.1 (26.6)	<.01	285	77.3 (32.4)	92.6 (11.6)	<.01	21	−21.6 (26.0)	235	0.3 (15.8)	<.01
Role functioning	654	75.9 (27.6)	33.4 (30.9)	<.01	285	49.6 (32.0)	83.9 (24.6)	<.01	21	−29.4 (38.3)	240	4.4 (32.4)	<.01
Emotional functioning	652	77.2 (20.4)	51.8 (28.5)	<.01	284	62.7 (32.1)	76.7 (21.7)	<.01	22	−11.7 (32.1)	237	12.1 (23.3)	<.01
Cognitive functioning	656	82.5 (20.7)	54.2 (28.8)	<.01	284	65.5 (32.1)	80.5 (22.7)	<.01	22	−17.4 (27.5)	240	6.9 (25.6)	<.01
Social functioning	648	81.9 (23.0)	52.3 (32.3)	<.01	283	66.9 (32.6)	81.3 (23.0)	<.01	21	−22.2 (42.9)	235	6.0 (29.7)	.01
Symptom scales													
Fatigue	656	28.2 (21.9)	59.5 (25.1)	<.01	284	44.9 (32.7)	25.9 (20.1)	<.01	22	22.2 (26.3)	240	−4.0 (24.4)	<.01
Nausea and vomiting	659	3.3 (9.0)	11.5 (19.4)	<.01	286	7.4 (32.3)	3.5 (9.7)	<.01	22	4.6 (23.7)	243	−2.3 (14.5)	.02
Pain	657	20.0 (25.2)	46.1 (32.6)	<.01	285	32.3 (32.6)	20.7 (25.6)	<.01	22	6.8 (38.4)	241	−5.1 (26.1)	.04
Dyspnea	657	14.9 (21.8)	30.6 (29.0)	<.01	286	23.9 (32.4)	12.2 (21.3)	<.01	22	12.1 (24.2)	242	0.7 (27.7)	.25
Insomnia	660	28.0 (29.2)	46.8 (35.7)	<.01	286	37.6 (32.8)	24.8 (30.0)	<.01	22	−3.0 (28.9)	244	−7.7 (32.4)	.61
Appetite loss	661	6.3 (16.2)	20.0 (27.6)	<.01	286	12.9 (32.2)	4.1 (15.2)	<.01	22	13.6 (36.6)	245	−0.5 (21.3)	<.01
Constipation	660	8.2 (18.8)	17.3 (26.4)	<.01	286	9.2 (32.9)	5.7 (18.7)	.05	22	10.6 (15.9)	244	−1.2 (24.8)	.02
Diarrhea	657	5.6 (14.8)	10.1 (21.4)	<.01	285	7.7 (32.6)	5.7 (14.6)	.32	22	3.0 (9.8)	241	−0.3 (20.0)	.37
Financial difficulties	654	10.4 (22.9)	27.1 (34.3)	<.01	283	25.4 (32.8)	11.4 (25.2)	<.01	22	18.2 (30.4)	238	0.1 (22.0)	<.01
EORTC QLQ-BN20													
Multi-item scales													
Future uncertainty	642	20.6 (19.2)	42.9 (24.3)	<.01	279	34.1 (32.9)	17.7 (17.5)	<.01	22	3.8 (5.2)	231	−10.9 (23.2)	.03
Visual disorder	651	10.6 (17.3)	25.6 (27.2)	<.01	282	18.4 (32.8)	12.1 (18.1)	.03	21	7.9 (33.0)	238	−2.9 (19.3)	<.01
Motor dysfunction	648	10.2 (15.5)	33.6 (30.6)	<.01	282	16.6 (32.1)	6.5 (13.1)	<.01	22	17.7 (31.9)	234	−7.0 (21.1)	<.01
Communication deficit	652	8.6 (13.8)	23.7 (25.5)	<.01	282	17.0 (32.9)	9.2 (15.1)	<.01	22	13.6 (30.7)	239	−3.0 (18.6)	.07
Single-item scales													
Headaches	656	21.8 (27.2)	41.4 (35.3)	<.01	284	38.0 (32.1)	28.0 (30.3)	.02	22	−7.6 (42.3)	241	−6.8 (30.2)	<.01
Seizures	652	2.5 (11.6)	7.6 (19.9)	<.01	282	4.7 (32.8)	3.3 (12.4)	.31	22	6.1 (22.2)	238	−3.9 (18.5)	.07
Drowsiness	656	27.8 (25.7)	55.5 (31.4)	<.01	284	43.1 (32.1)	30.5 (29.7)	<.01	22	22.7 (33.2)	241	−5.4 (28.8)	<.01
Hair loss	653	5.9 (17.0)	12.6 (28.1)	.01	284	10.4 (32.5)	5.7 (14.6)	.41	22	4.6 (23.7)	238	4.8 (24.0)	.72
Itchy skin	653	8.4 (18.3)	13.3 (24.2)	.02	284	8.9 (32.9)	8.5 (18.7)	.86	22	9.1 (25.6)	238	1.8 (22.5)	.42
Weakness of legs	649	9.1 (19.1)	25.9 (32.5)	<.01	283	11.9 (32.6)	2.0 (9.6)	<.01	22	9.1 (25.6)	235	−3.8 (24.8)	.03
Bladder control	656	9.3 (20.2)	26.5 (35.1)	<.01	284	14.7 (32.4)	5.7 (16.4)	.01	22	6.1 (28.4)	241	−5.5 (27.7)	.20

Abbreviations: QLQ-C30, Core Quality of Life Questionnaire; QLQ-BN20, brain cancer-specific Quality of Life Questionnaire; QoL, quality of life; WHO, World Health Organization; PS, performance status; Obs, observations; SD, standard deviation.

In the responsiveness analyses based on shifts or stability in WHO PS through surgery, significant differences were observed for all scales except Dyspnea, Insomnia, Diarrhea, Communication deficit, Seizures, Hair loss, Itchy skin, and Bladder control. For the Headaches scale, the mean score decreased, indicating a reduced level of symptomatology among patients with worsened WHO PS, while for Financial difficulties the mean score increased, indicating an increased experience of financial difficulties among patients with stable or improved WHO PS. For every other scale, patients with worsened WHO PS reported on average reduced health status/QoL and functioning, and an increased level of symptomatology (Table 5).

Correlations between scales of QLQ-C30 and scales of QLQ-BN20 are shown in Supplementary Table 1 (Appendix). All correlations except one (Seizures—Constipation) were statistically significant. However, correlation coefficients were with few exceptions higher for correlations between the functional and multi-item scales than for correlations involving symptom or single-item scales. As expected, some correlations between symptom scales were observed, for example, Fatigue—Drowsiness (|*r*| = 0.71), Pain—Headache (|*r*| = 0.56), Physical functioning—Weakness of legs (|*r*| = 0.53).

## Discussion

The EORTC questionnaires QLQ-C30 and QLQ-BN20 are among the most widely employed and advocated to assess HRQoL in patients with meningiomas but, prior to this report, had not undergone a disease-specific validation. While the instruments demonstrate high clinical and overall validity, there are limitations researchers need to be aware of when the questionnaires are applied to meningioma patients specifically. Several items display floor and ceiling effects, which may indicate a lack of sensitivity to levels of severity of specific functions and symptoms, and for which complementary measures are likely necessary. Also originally developed to study malignant brain tumors, some items were expected to be of limited relevance to patients with meningiomas but this seems an acceptable drawback considering the high response and completion rate found. Moreover, a high concordance of WHO PS assessments between 2 separate investigators was shown and suggests that WHO PS categorized binarily can be a useful measure with substantial interrater agreement when applied to patients with intracranial meningiomas.

There are several limitations to this study. First, although the number of unique observations (*n* = 767) greatly exceeded the stipulated 500 responses required, it could be argued that the number of unique *responders* did not meet the preset sample size. However, review of the statistical analyses and results does not indicate that lack of statistical power had a significant impact on the study, as the absolute majority of analyses and all clinical hypotheses reached formal statistical significance or were distinctly negative. Second, this is a single-center study using only the Swedish version of the EORTC questionnaires. Thus, cultural and linguistic diversity are not accounted for and may theoretically reduce the external validity of the results. However, previous validation studies on the EORTC QLQ-C30 and QLQ-BN20 that excluded patients with meningiomas nonetheless included culturally diverse samples of patients with brain cancer from several different countries, of which none reported issues in terms of generalizability within these domains,^5,8^ suggesting that translation and applicability across different languages and cultures would unlikely be of major concern for patients with meningiomas specifically. Third, due to administrative failure, a non-negligible number of patients responded to the preoperative form first after surgery but were, as a consequence, excluded from all analyses concerning preoperative scale scores. While this may, on the one hand, be considered a drawback, the figure is in line with a corresponding Scandinavian report on HRQoL in patients with diffuse gliomas using the EORTC instruments.^25^ Importantly, on the other hand, it highlights the current study’s population-based design, which is a significant strength that contrasts with similar validation reports that have been based on highly selected randomized controlled trials,^8^ or in which the patient recruitment process is not reported,^9–11,13^ resulting in significant limitations in terms of evaluating questionnaire acceptability and external validity. Furthermore, WHO PS was estimated by retrospective chart review exclusively. For most patients, scrutiny of medical records rendered sufficient information to determine functional status; however, medical notes were in some cases summary, thus resulting in estimations that were likely less accurate. Still, a substantial level of agreement between observers was demonstrated.

The population-based design is a major strength of the current study and shows that 9 out of 10 patients were willing to respond to the questionnaire. Although attitudes toward research participation may differ between different countries and cultures, this finding nevertheless indicates a high acceptability of the EORTC QLQ-C30 and QLQ-BN20 questionnaires among patients with intracranial meningiomas. The adequate sample size is also an important methodological strength and contrasts with most similar validation reports, wherein insufficient statistical power prevented firm and detailed conclusions to be drawn.^10–12^ Additionally, as previous studies have found varying interrater concordance in PS assessment between different diagnoses,^26^ an interrater reliability analysis of the WHO PS was included and showed a substantial level of agreement, thus substantiating its applicability. The subsequent use of WHO PS as a validated anchor in addition to data on sick leave to assess the clinical validity of the EORTC instruments adds to the robustness of the report.

Convergent and discriminant validity met the preset criteria for nearly all analyses, indicating an adequate representation and categorization of items into scales. Surprisingly, the only exception was the correlation between item 5 (“Do you need help with eating, dressing, washing yourself, or using the toilet?”) and the Physical functioning scale. While this may intuitively appear as a false-negative result, it could also reflect that the patients’ perception of capability of performing activities of daily living is not solely dependent on their physical functioning but also cognitive and mental functioning to a significant (and possibly even greater) extent.^27^ Moreover, except for Nausea and vomiting, the internal consistency was high for all multi-item scales, hence demonstrating high uniformity between items within the same multi-item scale. One possible explanation for the Nausea and vomiting internal inconsistency could be that these symptoms are addressed separately by the items included in the multi-item scale, and that several patients might have experienced nausea as an isolated symptom without vomiting. Another explanation could be that these symptoms are infrequent in patients with meningiomas, a notion further supported by the vast floor effects observed within these domains, whereas especially in contrast to malignant tumors, more often eligible for chemotherapy, and thus with a much higher likelihood of suffering from these symptoms.^28^ In addition, none of the scales evaluated demonstrated *very high* internal consistency (generally α > 0.95), which indicates that items supposed to evaluate a common construct managed to do so effectively by adding information rather than by replicating each other.

Results of the clinical validity analyses indicated a high level of concordance between the questionnaires and WHO PS and sick-leave status. The questionnaires were also responsive to changes in WHO PS, as scale score changes were in line with shifts in PS for most scales. These results altogether indicate that the questionnaires are susceptible to clinically and functionally important differences between patients, including their progression over time. For some symptom scales, however, score changes were not statistically significantly different between patients classified according to shifts in PS. Although functional deficits and specific symptoms are expected to correlate to some extent, the nonsignificant changes in mean scores observed for some specific symptoms may reflect that the WHO PS grading system primarily mirrors the patient’s overall functional status rather than the influence of certain symptoms. Furthermore, the mean score difference of the Physical functioning scale was surprisingly low (0.28) among patients with stable or improved WHO PS. However, since this group mostly composed of patients with stable WHO PS, few observations may be a possible explanation of this finding.

Several scales of the QLQ-C30 correlated considerably with those of QLQ-BN20. Some correlations were expected due to potential overlapping between certain scales, for example, Pain—Headache. However, correlations between more conceptually different scales were also observed, for example, Motor dysfunction—Cognitive functioning (|*r*| = 0.45), possibly reflecting a greater complexity of neurological deficits in patients experiencing these symptoms.

Although no adequately powered study has validated the QLQ-C30 and QLQ-BN20 questionnaires for meningioma patients specifically, the instruments have previously been evaluated in studies on patients with brain tumors,^8–13^ 2 of which included meningiomas.^10,13^ While our findings in general conform to the results of these studies, pronounced floor effects suggest some of the questionnaire items are likely of limited relevance when applied to patients with intracranial meningiomas specifically. As an expected observation, this item redundancy could nevertheless be viewed as an acceptable limitation considering the high response rate and completion rate of the questionnaires. However, item ceiling effects were also found and may indicate a lack of questionnaire sensitivity to the full range of severity within these domains specifically. Awareness of these limitations is thus required when the EORTC instruments are applied to meningioma patients, while supplemental analyses are likely necessary to be able to stratify between patients when these outcomes are of particular interest. Moreover, in contrast to several previous studies, the internal consistency for the Cognitive functioning scale met the preset threshold to be considered significant in the current report.^10,12,13,22^ This observation may reflect the higher internal validity of the present report wherein meningiomas were studied exclusively.

There are today several generic and brain tumor–specific questionnaires available to study patients with meningiomas, out of which the EORTC questionnaires have been considered one of the most appropriate to evaluate meningioma patients specifically.^2^ Nonetheless, there has also in line with the current study findings been increasing awareness that generic instruments may lack sensitivity to nuances of specific symptoms and functions,^7,29^ which have resulted in recent efforts to develop and validate meningioma-specific questionnaires.^7^ While these disease-specific questionnaires seem promising, healthy reference populations, cross-cultural validation, and translation across different languages are still lacking at this stage and may suggest that the EORTC questionnaires will likely continue to play an important role in studies on meningiomas. Importantly, however, researchers need to be aware of strengths of limitations of different questionnaires already at the time of study conceptualization and design in order to be able tailor the study approach to the research question at hand, and for which the current study aims to provide sufficient details for evaluation of the EORTC instruments for studies on meningiomas specifically.

In conclusion, the present study shows that the EORTC QLQ-C30 and QLQ-BN20 questionnaires are generally valid instruments to assess HRQoL and symptoms in patients with intracranial meningiomas but require awareness of possible limitations when specific functions and symptoms are evaluated. In addition, WHO PS assessment based on chart review had substantial interrater agreement when patients were categorized as either having a high or a low PS, suggesting that dichotomized WHO PS could be a useful measure in studies on patients with intracranial meningiomas.

## Supplementary material

Supplementary material is available online at *Neuro-Oncology Practice* (https://academic.oup.com/nop/).

npae125_suppl_Supplementary_Table_1

## Data Availability

Upon reasonable request, data and methodology, including STATA software code, can be shared. However, access to such data will be subject to external review by the Swedish Ethical Review Board and Umeå University concerning data sharing according to the European Union general data protection regulation (GDPR).

## Permission to reproduce material from other sources

No material from other sources was included in this article.

## References

[1] Goldbrunner R , Stavrinou P, Jenkinson MD, et al EANO guideline on the diagnosis and management of meningiomas. Neuro Oncol. 2021;23(11):1821–1834. 34181733 10.1093/neuonc/noab150 PMC8563316

[2] Zamanipoor Najafabadi AH , Peeters MCM, Dirven L, et al Impaired health-related quality of life in meningioma patients—a systematic review. Neuro Oncol. 2017;19(7):897–907. 28039363 10.1093/neuonc/now250 PMC5570251

[3] Nassiri F , Price B, Shehab A, et al Life after surgical resection of a meningioma: a prospective cross-sectional study evaluating health-related quality of life. Neuro Oncol. 2019;21(Supplement_1):i32–i43. 30649488 10.1093/neuonc/noy152 PMC6347082

[4] Zamanipoor Najafabadi AH , van der Meer PB, Boele FW, et al; Dutch Meningioma Consortium. Long-term disease burden and survivorship issues after surgery and radiotherapy of intracranial meningioma patients. Neurosurgery. 2020;88(1):155–164. 32818258 10.1093/neuros/nyaa351 PMC7735868

[5] Aaronson NK , Ahmedzai S, Bergman B, et al The European Organization for Research and Treatment of Cancer QLQ-C30: a quality-of-life instrument for use in international clinical trials in oncology. J Natl Cancer Inst. 1993;85(5):365–376. 8433390 10.1093/jnci/85.5.365

[6] Osoba D , Aaronson NK, Muller M, et al The development and psychometric validation of a brain cancer quality-of-life questionnaire for use in combination with general cancer-specific questionnaires. Qual Life Res. 1996;5(1):139–150. 8901377 10.1007/BF00435979

[7] Baba A , Saha A, McCradden MD, et al Development and validation of a patient-centered, meningioma-specific quality-of-life questionnaire. J Neurosurg. 2021;135(6):1685–1694. 33990085 10.3171/2020.11.JNS201761

[8] Taphoorn MJ , Claassens L, Aaronson NK, et al; EORTC Quality of Life Group, and Brain Cancer, NCIC and Radiotherapy Groups. An international validation study of the EORTC brain cancer module (EORTC QLQ-BN20) for assessing health-related quality of life and symptoms in brain cancer patients. Eur J Cancer (Oxford, England: 1990). 2010;46(6):1033–1040. 10.1016/j.ejca.2010.01.012 20181476

[9] Khoshnevisan A , Yekaninejad MS, Ardakani SK, et al Translation and validation of the EORTC brain cancer module (EORTC QLQ-BN20) for use in Iran. Health Qual Life Outcomes. 2012;10(1):54. 22607028 10.1186/1477-7525-10-54 PMC3422187

[10] Shin YS , Kim JH. Validation of the Korean version of the European Organization for Research and Treatment of Cancer brain cancer module (EORTC QLQ-BN20) in patients with brain tumors. Health Qual Life Outcomes. 2013;11(1):145. 23981813 10.1186/1477-7525-11-145 PMC3766694

[11] Zhang K , Tian J, He Z, et al Validation of the Chinese version of EORTC QLQ-BN20 for patients with brain cancer. Eur J Cancer Care (Engl). 2018;27(2):e12832. 29461664 10.1111/ecc.12832

[12] Cacho-Díaz B , Lorenzana-Mendoza NA, Oñate-Ocaña LF. Quality of life in brain cancer: clinical validation of the Mexican-Spanish version of the EORTC QLQ-BN20 Questionnaire. Front Neurol. 2019;10:40. 30761074 10.3389/fneur.2019.00040 PMC6363944

[13] Zahid N , Martins RS, Zahid W, et al Translation and validation of the Urdu version of the European Organization for Research and Treatment of Cancer Core Quality of Life Questionnaire (EORTC QLQ-C30) and brain module (QLQ-BN20) in primary brain tumor patients. J Patient Rep Outcomes. 2021;5(1):79. 34487251 10.1186/s41687-021-00354-6 PMC8421474

[14] Keshwara SM , Gillespie CS, Mustafa MA, et al Quality of life outcomes in incidental and operated meningiomas (QUALMS): a cross-sectional cohort study. J Neurooncol. 2023;161(2):317–327. 36525165 10.1007/s11060-022-04198-y PMC9756745

[15] Myers J , Gardiner K, Harris K, et al Evaluating correlation and interrater reliability for four performance scales in the palliative care setting. J Pain Symptom Manage. 2010;39(2):250–258. 20152588 10.1016/j.jpainsymman.2009.06.013

[16] Sørensen JB , Klee M, Palshof T, Hansen HH. Performance status assessment in cancer patients. An inter-observer variability study. Br J Cancer. 1993;67(4):773–775. 8471434 10.1038/bjc.1993.140 PMC1968363

[17] Manuals | EORTC – Quality of Life. Updated 2017-07-13. https://qol.eortc.org/manuals/. Accessed March 14, 2022.

[18] Tsang S , Royse CF, Terkawi AS. Guidelines for developing, translating, and validating a questionnaire in perioperative and pain medicine. Saudi J Anaesth. 2017;11(Suppl 1):S80–S89. 28616007 10.4103/sja.SJA_203_17 PMC5463570

[19] Hughes DJ. Psychometric validity: establishing the accuracy and appropriateness of psychometric measures. In Irwing P, Booth T, Hughes DJ eds. *The Wiley handbook of psychometric testing: A multidisciplinary reference on survey, scale and test development*. Wiley Blackwell; 2018:751–779.

[20] Cohen J. A coefficient of agreement for nominal scales. Educ Psychol Meas. 2024;20(1):37–46.

[21] Landis JR , Koch GG. The measurement of observer agreement for categorical data. Biometrics. 1977;33(2):363–374. 843571

[22] Oñate-Ocaña LF , Alcántara-Pilar A, Vilar-Compte D, et al Validation of the Mexican Spanish version of the EORTC C30 and STO22 questionnaires for the evaluation of health-related quality of life in patients with gastric cancer. Ann Surg Oncol. 2009;16(1):88–95. 18979141 10.1245/s10434-008-0175-9

[23] McHorney CA , Tarlov AR. Individual-patient monitoring in clinical practice: are available health status surveys adequate? Qual Life Res 1995;4(4):293–307. 7550178 10.1007/BF01593882

[24] Fayers PM , Machin D. Quality of Life: *The Assessment, Analysis and Interpretation of Patient-Reported Outcomes*. Third edition. Oxford: John Wiley & Sons Inc; 2016.

[25] Schei S , Solheim O, Salvesen O, Hansen TI, Sagberg LM. Patient-reported cognitive function before and after glioma surgery. Acta Neurochir. 2022;164(8):2009–2019. 35668303 10.1007/s00701-022-05261-3 PMC9338128

[26] Chow R , Zimmermann C, Bruera E, et al Inter-rater reliability in performance status assessment between clinicians and patients: a systematic review and meta-analysis. BMJ Support Palliat Care. 2020;10(2):129–135. 10.1136/bmjspcare-2019-002080 31806655

[27] Edemekong PF , Bomgaars DL, Sukumaran S, Schoo C. Activities of Daily Living. Treasure Island, FL: StatPearls Publishing; 2023. 29261878

[28] Liu R , Page M, Solheim K, Fox S, Chang SM. Quality of life in adults with brain tumors: current knowledge and future directions. Neuro Oncol. 2009;11(3):330–339. 19001097 10.1215/15228517-2008-093 PMC2718978

[29] Zamanipoor Najafabadi AH , Peeters MCM, Lobatto DJ, et al Health-related quality of life of cranial WHO grade I meningioma patients: are current questionnaires relevant? Acta Neurochir. 2017;159(11):2149–2159. 28952044 10.1007/s00701-017-3332-8 PMC5636848

