# Supplementary material for: Validation of the EORTC QLQ-C30 and QLQ-BN20, including WHO performance status interrater reliability, for evaluation of patients with intracranial meningiomas
Source: Neurooncol Pract. 2024 Dec 24;12(3):467–77. doi: 10.1093/nop/npae125 (PMC12137214; doi:10.1093/nop/npae125)
Supplement: npae125_suppl_Supplementary_Table_1 [file npae125_suppl_supplementary_table_1.docx]

| Supplemental Table 1 - Construct validity | | | | | | |  |  |  |  |  |  |  |  |  |  |  |  |  |  |  |  |  |
| --- | --- | --- | --- | --- | --- | --- | --- | --- | --- | --- | --- | --- | --- | --- | --- | --- | --- | --- | --- | --- | --- | --- | --- |
|  |  | ***Multi-item scales*** | | |  |  |  |  | ***Single-item scales*** | | | | |  |  |  |  |  |  |  |  |  |  |
|  |  | **FU** | | **VD** | | **MD** | | **CD** | | **He** | | **Se** | | **Dr** | | **HL** | | **IS** | | **WoL** | | **BC** | |
|  |  | **R** | ***p*** | **R** | ***p*** | **R** | ***p*** | **R** | ***p*** | **R** | ***p*** | **R** | ***p*** | **R** | ***p*** | **R** | ***p*** | **R** | ***p*** | **R** | ***p*** | **R** | ***p*** |
|  | **GH/QoL** | -0.66 | <0.01 | -0.39 | <0.01 | -0.54 | <0.01 | -0.44 | <0.01 | -0.38 | <0.01 | -0.17 | <0.01 | -0.54 | <0.01 | -0.18 | <0.01 | -0.15 | <0.01 | -0.42 | <0.01 | -0.30 | <0.01 |
| *Functional scales* | | | |  |  |  |  |  |  |  |  |  |  |  |  |  |  |  |  |  |  |  |  |
|  | **PF** | -0.49 | <0.01 | -0.34 | <0.01 | -0.59 | <0.01 | -0.37 | <0.01 | -0.24 | <0.01 | -0.17 | <0.01 | -0.50 | <0.01 | -0.27 | <0.01 | -0.24 | <0.01 | -0.53 | <0.01 | -0.32 | <0.01 |
|  | **RF** | -0.58 | <0.01 | -0.41 | <0.01 | -0.53 | <0.01 | -0.40 | <0.01 | -0.33 | <0.01 | -0.17 | <0.01 | -0.51 | <0.01 | -0.17 | <0.01 | -0.17 | <0.01 | -0.40 | <0.01 | -0.23 | <0.01 |
|  | **EF** | -0.69 | <0.01 | -0.35 | <0.01 | -0.42 | <0.01 | -0.41 | <0.01 | -0.40 | <0.01 | -0.21 | <0.01 | -0.50 | <0.01 | -0.11 | <0.01 | -0.10 | <0.01 | -0.29 | <0.01 | -0.22 | <0.01 |
|  | **CF** | -0.53 | <0.01 | -0.57 | <0.01 | -0.45 | <0.01 | -0.60 | <0.01 | -0.41 | <0.01 | -0.20 | <0.01 | -0.51 | <0.01 | -0.16 | <0.01 | -0.18 | <0.01 | -0.34 | <0.01 | -0.24 | <0.01 |
|  | **SF** | -0.60 | <0.01 | -0.35 | <0.01 | -0.50 | <0.01 | -0.39 | <0.01 | -0.35 | <0.01 | -0.17 | <0.01 | -0.52 | <0.01 | -0.17 | <0.01 | -0.16 | <0.01 | -0.36 | <0.01 | -0.24 | <0.01 |
| *Symptom scales* | | | |  |  |  |  |  |  |  |  |  |  |  |  |  |  |  |  |  |  |  |  |
|  | **Fa** | 0.57 | <0.01 | 0.45 | <0.01 | 0.56 | <0.01 | 0.41 | <0.01 | 0.44 | <0.01 | 0.17 | <0.01 | 0.71 | <0.01 | 0.21 | <0.01 | 0.22 | <0.01 | 0.46 | <0.01 | 0.24 | <0.01 |
|  | **NV** | 0.27 | <0.01 | 0.24 | <0.01 | 0.24 | <0.01 | 0.22 | <0.01 | 0.31 | <0.01 | 0.11 | <0.01 | 0.25 | <0.01 | 0.14 | <0.01 | 0.12 | <0.01 | 0.2 | <0.01 | 0.11 | <0.01 |
|  | **Pa** | 0.47 | <0.01 | 0.35 | <0.01 | 0.36 | <0.01 | 0.29 | <0.01 | 0.56 | <0.01 | 0.17 | <0.01 | 0.50 | <0.01 | 0.20 | <0.01 | 0.18 | <0.01 | 0.32 | <0.01 | 0.17 | <0.01 |
|  | **Dy** | 0.29 | <0.01 | 0.25 | <0.01 | 0.35 | <0.01 | 0.33 | <0.01 | 0.24 | <0.01 | 0.08 | 0.02 | 0.35 | <0.01 | 0.17 | <0.01 | 0.19 | <0.01 | 0.35 | <0.01 | 0.24 | <0.01 |
|  | **In** | 0.42 | <0.01 | 0.28 | <0.01 | 0.34 | <0.01 | 0.24 | <0.01 | 0.29 | <0.01 | 0.15 | <0.01 | 0.37 | <0.01 | 0.10 | <0.01 | 0.14 | <0.01 | 0.26 | <0.01 | 0.20 | <0.01 |
|  | **AL** | 0.32 | <0.01 | 0.26 | <0.01 | 0.27 | <0.01 | 0.25 | <0.01 | 0.24 | <0.01 | 0.13 | <0.01 | 0.27 | <0.01 | 0.09 | 0.02 | 0.12 | <0.01 | 0.24 | <0.01 | 0.13 | <0.01 |
|  | **Co** | 0.21 | <0.01 | 0.18 | <0.01 | 0.28 | <0.01 | 0.25 | <0.01 | 0.16 | <0.01 | 0.05 | 0.16 | 0.21 | <0.01 | 0.11 | <0.01 | 0.12 | <0.01 | 0.26 | <0.01 | 0.19 | <0.01 |
|  | **Di** | 0.13 | <0.01 | 0.17 | <0.01 | 0.18 | <0.01 | 0.14 | <0.01 | 0.12 | <0.01 | 0.13 | <0.01 | 0.17 | <0.01 | 0.09 | 0.02 | 0.09 | 0.02 | 0.16 | <0.01 | 0.15 | <0.01 |
|  | **FD** | 0.41 | <0.01 | 0.25 | <0.01 | 0.20 | <0.01 | 0.30 | <0.01 | 0.29 | <0.01 | 0.16 | <0.01 | 0.37 | <0.01 | 0.10 | <0.01 | 0.08 | 0.04 | 0.2 | <0.01 | 0.11 | <0.01 |
| Abbreviations: R, Spearman's rho; GH, Global health status; QoL, Quality of Life; PF, Physical functioning; RF, Role functioning; EF, Emotional functioning; CF, Cognitive functioning; SF, Social functioning; Fa, Fatigue; NV, Nausea and vomiting; Pa, Pain; Dy, Dyspnoea; In, Insomnia; AL, Appetite loss; Co, Constipation; Di, Diarrhoea; FD, Financial difficulties; FU, Future uncertainty; VD, Visual disorder; MD, Motor dysfunction; CD, Communication deficit; He, Headaches; Se, Seizures; Dr, Drowsiness; HL, Hair loss; IS, Itchy skin; WoL, Weakness of legs; BC, Bladder control. | | | | | | | | | | | | | | | | | | | | | | | |
